# Uncommon Anatomical Variation of the Facial Artery: A Cadaveric Case Report

**DOI:** 10.7759/cureus.50275

**Published:** 2023-12-10

**Authors:** Georgios Langas, Stavros Tsiakaras, Ioannis Mykoniatis, Paraskevi Karamitsou, George K Paraskevas, Nikolaos Lazaridis, Chrysanthos Chrysanthou, Nikolaos Anastasopoulos, Alexandros Poutoglidis

**Affiliations:** 1 1st Department of Urology, Aristotle University of Thessaloniki, School of Medicine, 'G. Gennimatas' General Hospital, Thessaloniki, GRC; 2 Department of Otorhinolaryngology-Head and Neck Surgery, 'G. Papanikolaou" General Hospital, Thessaloniki, GRC; 3 Department of Anatomy and Surgical Anatomy, School of Medicine, Aristotle University of Thessaloniki, Thessaloniki, GRC

**Keywords:** cadaveric specimen, cadaver dissection, anatomical variability, anatomy dissection, facial artery

## Abstract

The facial artery is a branch of the external carotid artery, one of the major arteries supplying blood to the head and neck. The normal route of the facial artery follows a well-defined path. It typically arises from the external carotid artery, above the superior border of the hyoid bone. During its route, the facial artery gives off branches in the neck, mandible, buccal region, and face. This case report explores a rare anatomical variation of the facial artery characterized by an unusual termination point above the upper lip as the superior labial artery, found during a routine cadaveric dissection. While variations in the course of the facial artery are documented, this particular deviation, with its termination anterior to the typical endpoint, presents a unique anatomical variation.

## Introduction

The facial artery is a major branch of the external carotid artery, responsible for supplying blood to the structures of the face. Its standard course involves ascending from the carotid triangle, crossing the mandible, and subsequently branching into the superior and inferior labial arteries [1,2]. It terminates by anastomosing with branches of the ophthalmic artery and the contralateral facial artery at the medial angle of the eye, eventually forming the angular artery [2]. While the general course and branching pattern of the facial artery are well-documented, anatomical variations are not uncommon and may have implications for surgical procedures in the facial region. Exploring and comprehending the variability of diverse patterns of onset and termination of the facial artery holds considerable clinical significance in everyday practice. Six types of termination have been reported as follows: a. termination as an angular artery, b. termination as a lateral nasal artery, c. termination as an interior alar artery, d. terminating as a superior labial artery, e. termination as an inferior labial artery, and f. hypoplastic facial artery [1-3].

## Case presentation

During a routine cadaveric dissection performed at the Department of Anatomy and Surgical Anatomy of the School of Medicine of Aristotle University of Thessaloniki in Greece, a relatively uncommon variation of the facial artery was identified. The cadaver utilized for this study was of an 80-year-old male from the Balkan population and was donated to the School of Medicine of Aristotle University of Thessaloniki for educational purposes. No documented history of facial trauma, surgery, or congenital anomalies was available.

**Figure 1:**
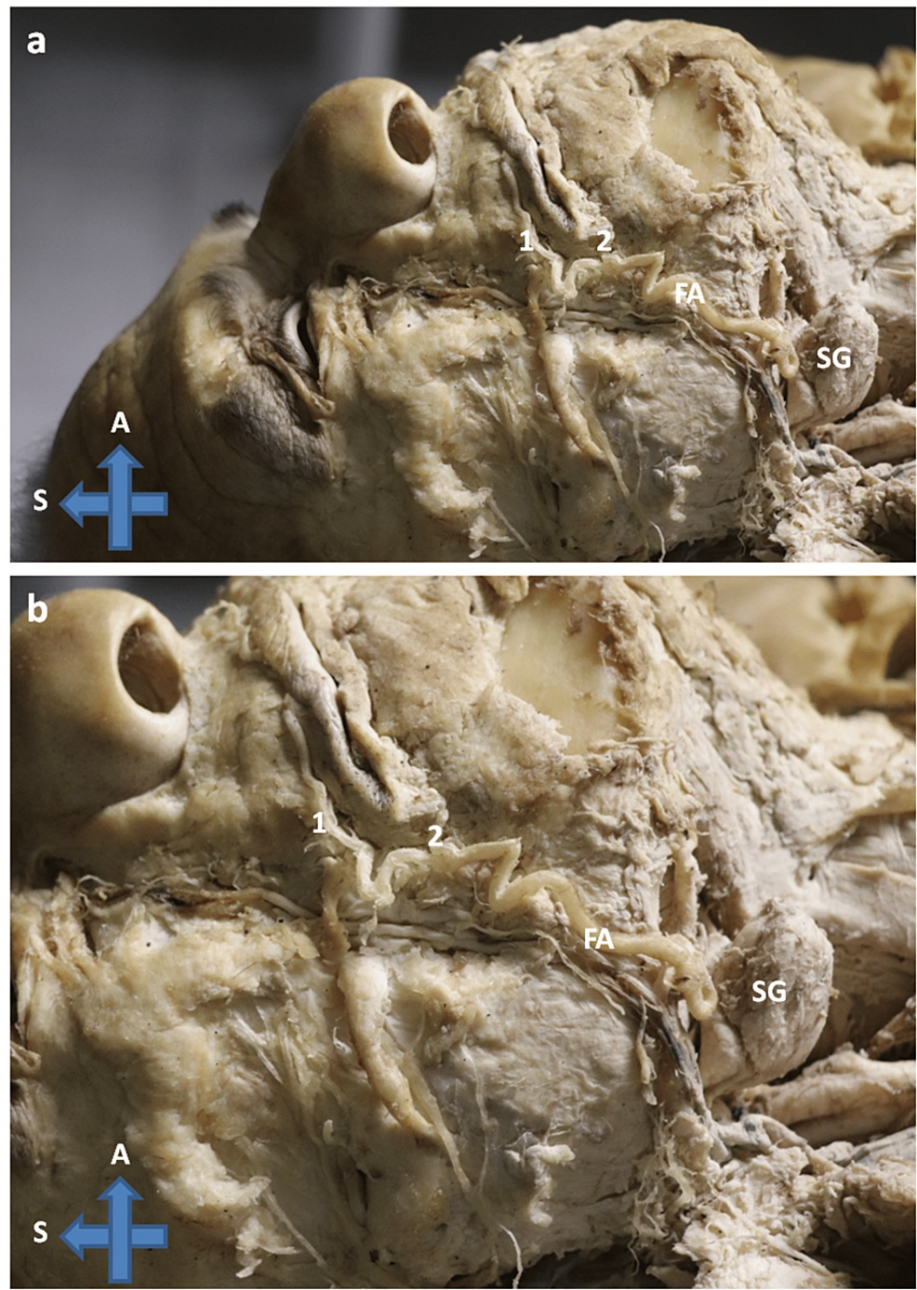
The course of the facial artery The course of the facial artery with the superior labial artery being its terminal segment S: superior, A: anterior, FA: facial artery, 1: superior labial artery, 2: inferior labial artery, SG: submandibular gland

The facial vasculature was thoroughly dissected to explore the course of the facial artery. In the standard anatomical configuration, the facial artery typically arises from the external carotid artery, ascends over the inferior border of the mandible, and follows a consistent course toward the angle of the mouth. During this dissection, a remarkable deviation from the conventional anatomical pathway was observed - an early termination of the facial artery above the upper lip as the superior labial artery (Figure 1).

The variation was unilateral, affecting the right side of the face. Due to this unusual termination, the facial artery did not give off its last branch, the angular artery, which forms as a result of the anastomosis of both facial arteries and the ophthalmic artery.

## Discussion

The termination of the facial artery above the upper lip has notable clinical implications. This anatomical variation challenges conventional surgical approaches in the mid-facial region, potentially affecting procedures such as cleft lip repair, maxillofacial surgery, and certain plastic surgery interventions. Our observations align with existing literature that emphasizes the importance of recognizing and understanding facial artery variations for clinical practitioners. The utility of vascular imaging is crucial in understanding such facial arterial variants [4]. Preoperative angiography or high-resolution computed tomography (CT) scans can play a pivotal role in identifying such anomalies, allowing surgeons to tailor their approaches accordingly. Previous literature underscores the variability in facial arterial anatomy and emphasizes the potential impact on surgical and interventional procedures [3]. Surgeons have to be aware of such anatomical variations to avoid inadvertent complications during mid-face procedures. Furthermore, Hong et al. assessed the diverse branching pattern of the facial artery and its branches through conventional angiography [5]. A facial artery termination as a superior labial artery was shown in 8.5% of a total of 284 angiographies [5]. Similarly to these findings, a study conducted in 2020 analyzed 284 hemifaces obtained from 142 cadavers preserved in formalin [6]. The researchers noted that 8.4% of dissected facial arteries terminated as superior labial arteries [6]. The deviation observed prompts a re-evaluation of the vascular supply to the structures around the upper lip. In a meta-analysis conducted in 2021 by Koziej et al., the incidence of termination on either the superior or inferior labial artery was documented to be 15.5% [2]. In a study by Lohn et al., which delved into the trajectory, branching configurations, terminations, and atypical variations of the facial artery and vein, cadaveric dissections involving 201 facial arteries and 198 facial veins were executed [3]. The findings revealed a prevalence of 10% for superior labial termination.

Understanding the variations in arterial anatomy of the head and neck region is vital for preventing complications related to ischemia or other during surgical interventions [7,8]. This case underscores the importance of incorporating anatomical variations into medical education curricula. Increased awareness among medical students and practitioners is crucial for improving patient safety and the efficacy of medical interventions.

## Conclusions

This cadaveric study unveils a relatively uncommon anatomical variation of the facial artery - a termination above the upper lip in a male specimen. The detailed exploration of this anomaly sheds light on its clinical implications and underscores the importance of awareness and adaptability in surgical and medical practices. As anatomical variations continue to be uncovered, ongoing research in this field remains of paramount importance for advancing our understanding of human anatomy and optimizing patient care.

## References

[1] Koh KS, Kim HJ, Oh CS, Chung IH (2003). Branching patterns and symmetry of the course of the facial artery in Koreans. Int J Oral Maxillofac Surg.

[2] Koziej M, Bonczar M, Ostrowski P (2022). Termination points of the facial artery—a meta-analysis. Clin Anat.

[3] Lohn JW, Penn JW, Norton J, Butler PE (2011). The course and variation of the facial artery and vein: implications for facial transplantation and facial surgery. Ann Plast Surg.

[4] Alharbi YA (2023). Anatomical study of divergences in facial artery endings. Anat Cell Biol.

[5] Hong SJ, Park SE, Jo JW (2020). Variant facial artery anatomy revisited: conventional angiography performed in 284 cases. Medicine (Baltimore).

[6] Loukas M, Hullett J, Louis RG Jr, Kapos T, Knight J, Nagy R, Marycz D (2006). A detailed observation of variations of the facial artery, with emphasis on the superior labial artery. Surg Radiol Anat.

[7] Nakajima H, Imanishi N, Aiso S (2002). Facial artery in the upper lip and nose: anatomy and a clinical application. Plast Reconstr Surg.

[8] Poutoglidis A, Savvakis S, Karamitsou P (2023). Is the origin of the superior thyroid artery consistent? A systematic review of 5488 specimens. Am J Otolaryngol.

